# Gamete and Embryo Donation and Surrogacy in Australia: The Social Context and Regulatory Framework 

**Published:** 2011-02-20

**Authors:** Karin Hammarberg, Louise Johnson, Tracey Petrillo

**Affiliations:** 1 Melbourne School of Population Health, The University of Melbourne, Melbourne, Australia; 2 Victorian Assisted Reproductive Treatment Authority, Melbourne, Australia

**Keywords:** Gamete Donation, Surrogate Mothers, Legal Aspects, Psychosocial Aspects, Review

## Abstract

The social and legal acceptability of third-party reproduction varies around the world. In Australia, gamete and embryo donation and surrogacy are permitted within the regulatory framework set out by federal and state governments. The aim of this paper is to describe the social context and regulatory framework for third-party reproduction in Australia.

This is a review of current laws and regulations related to third-party reproduction in Australia. Although subtle between-state differences exist, third-party reproduction is by and large a socially acceptable and legally permissible way to form a family throughout Australia. The overarching principles that govern the practice of third-party reproduction are altruism; the right of donorconceived people to be informed of their biological origins; and the provision of comprehensive counselling about the social, psychological, physical, ethical, financial and legal implications of third-party reproduction to those considering donating or receiving gametes or embryos and entering surrogacy arrangements. These principles ensure that donors are not motivated by financial gain, donor offspring can identify and meet with the person or persons who donated gametes or embryos, and prospective donors and recipients are aware of and have carefully considered the potential consequences of third-party reproduction.

Australian state laws and federal guidelines prohibit commercial and anonymous third-party reproduction; mandate counselling of all parties involved in gamete and embryo donation and surrogacy arrangements; and require clinics to keep records with identifying and non- identifying information about the donor/s to allow donor-conceived offspring to trace their biological origins.

## Introduction

Assisted reproductive technologies (ART) offer those who are affected by infertility an opportunity to have children. Most ART procedures involve heterosexual couples using their own oocytes and sperm to form embryos in the hope of having a child who is genetically linked to both partners. Couples where either the male or the female partner is affected by conditions that preclude the use of their own gametes may consider using donated gametes in ART procedures.

The need for donor sperm may arise due to lack of sperm production caused by genetic or environmental factors such as chemotherapy or radiation therapy, and some use donor sperm to avoid transmission of a genetic disease. Donor insemination (DI), where the woman in couples with untreatable male infertility is inseminated with donor sperm, has been a treatment option for several decades. Donor sperm can also be used in *in vitro* fertilisation (IVF) if the female partner also has a fertility problem. In more recent years, single women and lesbian couples have been able to use DI and IVF with donor sperm to have children in some countries. Donor oocytes may be used by couples where ovarian failure or declining ovarian function is the cause of infertility, poor oocyte quality has been identified in previous ART cycles, or the woman is a carrier of a severe genetic condition (1). Furthermore, couples who experience repeated treatment failure may be advised that their chance of becoming parents is higher if they use donor oocytes. For couples where both the woman and the man have problems relating to gamete production, donor embryos may be a treatment option. The use of donor oocytes and embryos to conceive has become increasingly common since this became technically possible some 25 years ago. In a small proportion of infertile couples the woman is unable to carry a pregnancy and in order to have a child they need to commission a surrogate to carry the pregnancy and give birth.

This paper discusses the social context and outlines the regulatory framework for gamete/embryo donation and surrogacy in Australia.

### The Commonwealth of Australia

The Commonwealth of Australia is a country in the southern hemisphere with a population of approximately 22 million people. Australia comprises six states: New South Wales (NSW), Queensland (Qld), South Australia (SA), Tasmania (Tas), Victoria (Vic) and Western Australia (WA) and two Territories: Australian Capital Territory (ACT) and the Northern Territory (NT). Legislation relating to health, including ART, is a matter for individual states.

Australia has a two-tiered health care system. The national universal taxpayer-funded Medicare and Pharmaceutical Benefit Schemes cover all Australians. Medicare allows access to out-of-hospital medical care and treatment in public hospitals at no or limited cost to the individual and the Pharmaceutical Benefit Scheme (PBS) reduces the personal cost of most prescribed medications. In addition to Medicare, private health insurance schemes which reduce the financial cost of private hospital care can be purchased.

### ART in Australia

ART has a long tradition in Australia where scientists and clinicians pioneered some of the ART techniques in the 1970’s and 1980’s (2). DI was introduced in 1970 (3), the first Australian IVF pregnancy was reported in 1981 (4), oocyte and embryo donation became treatment options in the mid 1980’s (5, 6), cryopreservation allowed surplus embryos to be frozen (7) and the first surrogacy birth in Australia was reported in 1988 (8). There is broad public acceptance of ART to treat infertility (9) in Australia and ART procedures are subsidised by Medicare and the PBS. As a result of these government subsidies, the utilisation rate of ART is higher and the financial cost of ART to couples is lower than in most other developed countries (10). Infertile couples can access affordable ART services at some 70 fertility clinics around Australia. Most ART procedures are performed in accredited fertility clinics but DI cycles can also be performed in hospitals and private practices. In 2007, approximately 52,000 ART treatment cycles were performed in Australian fertility clinics and over 10,000 children were born as a result, accounting for 3.1% of all Australian births that year (11). Of the treatment cycles performed in 2007, 1,800 were embryo transfers with donated oocytes or embryos resulting in 300 births, around 2,200 were DI cycles resulting in 250 births and 52 were surrogacy cycles resulting in 7 births (11). Donor procedures accounted for approximately 7.5% of all ART procedures in 2007. In addition, an unknown number of DI cycles were performed in hospitals and private clinics other than fertility clinics.

### Regulation of ART

There is no Australia-wide government body or legislation regulating the provision of ART services. However, all ART clinics are required to comply with the Code of Practice for Reproductive Technology Units developed by the Fertility Society of Australia’s Reproductive Technology Accreditation Committee (RTAC), which performs annual accreditation visits to all ART clinics (12). The purpose of the code is to set minimum standards for fertility clinics providing ART services in Australia and New Zealand and to encourage continuous improvement in the quality of care provided to people who undergo treatment. The RTAC Code of Practice requires all ART clinics in Australia and New Zealand to provide detailed information to the Australian Institute of Health and Welfare’s National Perinatal Statistics Unit (AIHW NPSU) about the number and type of treatment cycles performed and the number of pregnancies and births resulting from treatment. Treatment and pregnancy outcome data are compiled by the AIHW NPSU and published in an annual report (11).

The Australian Federal Government, through the National Health and Medical Research Council (NHMRC), has issued Ethical Guidelines on the Use of Assisted Reproductive Technology in Clinical Practice and Research (13) and RTAC requires fertility clinics to follow these.

In addition, four of the six Australian states have legislation which regulates ART in those states:

- The Assisted Reproductive Technology Act 2007 (NSW) and the associated Assisted Reproductive Technology Regulation 2009 in New South Wales (14)
- The Assisted Reproductive Treatment Act 1988 (SA) in South Australia (also adopted by the Northern Territory) (15)
- The Assisted Reproductive Treatment Act 2008 (Vic) and the associated Assisted Reproductive Treatment Regulations 2009 in Victoria (16, 17)
- The Human Reproductive Technology Act 1991 (WA) and the associated Human Reproductive Technology (Licences and Registers) Regulations 1993 in Western Australia (18).

### Gamete and embryo donation

#### Regulatory framework

In broad terms, State laws, the NHMRC Ethical Guidelines and the RTAC Code of Practice stipulate that:

- Only altruistic gamete and embryo donation is permissible. Apart from compensation for expenses incurred as a result of donating gametes or embryos, a donor cannot receive any payment or other inducement.
- Potential donors and recipients must receive adequate information about the medical, social, psychological and legal implications of donor procedures and counselling is mandatory before proceeding.
- Potential donors must undergo screening for infectious diseases.
- Recipients are entitled to access information about the donor’s medical history, physical characteristics, and the number and sex of children born from gametes donated by the same donor.
- Children born as a result of gamete or embryo donation have the right to access information about the donor, including identifying information.
- ART clinics are obliged to maintain detailed records, including identifying and non-identifying information, of donors, recipients and offspring which allow donor-conceived young adults to find out their genetic origins.
- In some states, ART clinics are obliged to provide information about donors, recipients and offspring to a central state register.
- The donor does not bear legal responsibilities for and is presumed not to be the parent of a child born as a result of his or her donation.
- A maximum number of families can be created with a donor’s gametes.

#### Informed decision making

There is agreement around the world that the social, emotional, medical, legal and ethical complexities of donor conception require thorough exploration by those donating and receiving gametes and embryos (19-23). The RTAC Code of Practice and the NHMRC Ethical Guidelines stipulate that individuals considering donor procedures receive counselling before they proceed.

The following matters are covered in donor counselling:

- Circumstances that lead to considering being a donor
- Medical and practical aspects of the procedure for the donor
- Psychological and social aspects of being a donor
- Legal aspects of being a donor including the possibility that a child who is born as a result of the donation may contact the donor in the future
- Possible impact of the donation on the donor’s relationship with his or her intimate partner
- Possible impact of the donation on the donor’s own children
- Possible impact of the donation on the donor’s relationship with the recipient if they are known to each other.

Counsellors also gauge prospective donors for their suitability to be a donor in terms of their medical and genetic history, personality characteristics and motivations for being a donor. People considering donating embryos are encouraged to contemplate their feelings about donating a potential full genetic sibling to their own child or children.

Counselling for recipients aims to ensure that they consider the implications of donor conception for themselves, a future child, their family and social networks, and, if the donor is known to them, the impact of the donation on their relationship with the donor.

The following matters are covered in recipient counselling:

- How a donor was found
- The lack of a genetic tie to one or both parents of a child born after a donor procedure
- Medical and practical aspects of the procedure for the recipient
- Psychological and social aspects of using a donor to conceive
- Legal aspects of using a donor to conceive
- Possible impact of using a donor to conceive on the intimate partner relationship
- Possible impact of the donation on the recipient’s relationship with the donor if they are known to each other
- The importance of disclosing the use of a donor to a child born as a result of gamete or embryo donation
- When, how and to whom to disclose the use of donor gametes or embryos
- Possible future interaction between the child and the donor.

In a prospective longitudinal survey of 72 donors and recipients in Victoria the majority perceived the counselling they received before undergoing donor procedures significantly more helpful than they had anticipated it would be. This suggests that counselling is beneficial for those contemplating donor procedures and helps potential donors and recipients become aware of the intricacies of donor conception (24).

#### Secrecy versus openness

In the past, parents were advised not to tell children that they were conceived using donated gametes (25, 26). However, over time attitudes have changed and openness is now recommended and encouraged. A contributing factor for this attitudinal shift is activism by donor-conceived adults and the Donor Conception Support Group (DCSG) in Australia. They argue that donor-conceived people have a fundamental right to know how they were conceived and to have access to information about their donor (27, 28). Parliamentary speeches in Victoria and New South Wales have also emphasised the rights of children to be able to access information about their donor and to be told about how their family was formed (28, 29).

The abolition of anonymous donation across Australia has undoubtedly also contributed to increased recognition of the needs and rights of donor-conceived people. Victoria was the first jurisdiction in the world to implement comprehensive legislation regulating the provision of ART services including the establishment of a state donor register (16, 30, 31). Donor-conceived young adults, their parents (on behalf of younger children) or donors can apply for information about each other through this register. Consent from the other party is required before information can be provided, with the exception of donor-conceived persons born under the conditions of the 1995 or 2008 legislation, whose donors were required to consent to the provision of identifying information prior to donating. In addition, voluntary registers enable those born prior to the introduction of legislation and those who wish to share information with other related parties to register information. This allows adult half-siblings, parents sharing the same donor or donor-conceived adults, their parents and donors to exchange information and meet if desired.

To increase the chance of donor-conceived people finding out about the way they were conceived, even if their parents do not disclose this to them, the recently introduced Victorian Assisted Reproductive Treatment Act 2008 (16) stipulates that from 2010, children born as a result of donor treatment have an addendum to their birth certificate informing them that additional information about their birth is available. It is expected that this addendum to donor-conceived persons’ birth certificates will be an incentive for parents to tell their children about their donor origins.

The capacity for donor-conceived adults or their parents to apply for information about their donor varies from state to state. In Western Australia, amendments to the Human Reproductive Technology Act 1991, allow donor-conceived adults to apply for information about their donors if they donated after December 2004 (18). In New South Wales, the Assisted Reproductive Technology Act 2007 makes provision for a central register to enable applications for information from donorconceived adults (14). In South Australia, amendments in 2009 to the Reproductive Technology (Clinical Practices) Act 1988 (now called the Assisted Reproductive Treatment Act 1988) include the establishment of a central register (15).

For states without legislation, guidelines now require clinics to only use donors who consent to the release of identifying information to children who are born as a result of their donation. From 2006, RTAC guidelines require fertility clinics across Australia to only accept donors who are prepared to provide identifying information to any offspring who wish to receive information about them on reaching adulthood and to have policies and procedures in place to support the offspring’s right to know their genetic origins (12). This echoes NHMRC Ethical Guidelines which state that "persons conceived using ART procedures are entitled to know their genetic parents" (13). However, clinics do not have the powers of government or statutory authorities to trace current contact details for donors to enable donor-conceived adults to find their donor. Therefore, in the absence of a central register in each state or at a national level, the rights of donor-conceived adults to obtain information about their donor remains unequal throughout Australia. The use of non-commercial donors from other countries by some Australian clinics may also present challenges in the future for donor-conceived adults who wish to find their donor 18 years or more after the donation.

Parents of donor-conceived children and donorconceived adults who are unable to access information about their donor can now use the Internet to search for half-siblings and the donor. In a recent study, which included participants from Australia, 73% of 791 parents of donor-conceived children who were members of the Internet-based Donor Sibling Registry (DSR) had found siblings and 18% had found the donor using the DSR website (32).

Australian research shows that although teenagers understand that it may be difficult for parents to ‘disclose such information,’ they believe parents who have used a donor to conceive should share this information with their children (33). Though openness is strongly advocated and parents are aware that information about the donor is recorded in clinics and some state registers, some parents find it difficult or are unwilling to disclose the use of a donor and many parents never tell their children they were donor-conceived. A study conducted in Victoria, following the introduction of the Infertility Treatment Act 1995 (31), found that only 37% of families had told their children about their conception, despite the legislated right for these children to apply for identifying information about their donor on reaching 18 years of age (34). However, a recent Australian study suggests that attitudes among parents are changing towards increased willingness to tell children about the way they were conceived (24). In this study, 77% of those considering using a donor to conceive stated that they felt positive about disclosing the use of a donor to the child.

Since 2006, the Victorian Infertility Treatment Authority (now the Victorian Assisted Reproductive Treatment Authority) has provided advice to parents on ‘how to tell’ children that the family was formed through the use of a donor. While health care professionals such as counsellors in clinics may have encouraged openness, parents are often anxious about what to say and how their child will react, and fear that their child may reject them when they find out that they are donor-conceived. Practical resources such as children’s books, podcasts of other families who share their experiences, seminars for parents and web-based and written information assist parents in talking to their children and help to increase their confidence. In practice, parents often report that telling their child or young adult about the way he or she was conceived was easier than they had anticipated and are relieved once they have shared the information (personal communication with Kate Bourne, Community Education Officer, Victorian Assisted Reproductive Treatment Authority).

#### Availability of gamete and embryo donors

Donors may be recruited by the ART clinic and be unknown to the recipient or recruited by the recipient and known and, in some instances, related to the recipient.

Most sperm donors are recruited by ART clinics through advertising campaigns and public awareness activities. Some clinics have a sufficient number of sperm donors to meet demand. However, the requirement to only use donors who are willing to provide identifying information to any offspring and broadened access to treatment, to include single women and same-sex couples in certain states, has led to a shortage of sperm donors in some clinics. Occasionally, couples recruit their own sperm donor, either through social networks or advertising.

Occasionally oocyte donors are recruited by ART clinics and are unknown to the recipient couple. However, due to the shortage of women who are prepared to donate oocytes to someone they do not know, most recipient couples rely on a friend or a relative to agree to donate oocytes, or attempt to recruit an oocyte donor through advertisements in local newspapers or via the Internet.

There is a substantial shortage of embryos available for donation. At the end of the legal storage time limit for frozen embryos (five years in most states), couples are required to decide the fate of frozen embryos that they are not intending to use. Most couples donate these embryos to research or choose to have them discarded and only 10-15% of couples donate their embryos to another infertile couple (35, 36). Common reasons for discarding rather than donating frozen embryos is the perception of the embryo as a potential child and sibling to existing children and the risk of being contacted by a child born as a result of the donation in the future (35, 37). It has been suggested that education programs and encouraging couples to reflect on their beliefs about the importance of genetic relatedness may increase the number of couples who donate their surplus embryos to another infertile couple (36, 38).

#### Eligibility criteria for access to ART treatment

Eligibility requirements for access to ART services vary throughout Australia. In New South Wales, Victoria and Western Australia, access to ART services is broad, enabling any woman, regardless of relationship status or sexual orientation to have ART treatment (14, 16, 18). Hence, in these states single women and lesbian couples can use donor sperm or embryos in ART procedures. South Australian law restricts access to ART to heterosexual married or de facto couples and single women who are medically infertile, whereas lesbian couples and single women who are not medically infertile are denied access (15, 39). In all other states, eligibility for ART treatment is determined by individual clinics.

ART legislation has been challenged in the courts in South Australia and Victoria (39, 40). In both these cases the plaintiff contended that eligibility requirements for access to treatment in these states were discriminatory. The courts supported these claims and in each case declared the relevant state legislation to be inconsistent with Australia’s Sex Discrimination Act 1984 (Cth) (41). As a result, the administration of legislation in South Australia and Victoria changed to allow treatment for medically infertile single women and lesbian couples. Since then, the Victorian legislation has been revised and now also allows access to ART treatment for fertile lesbian couples and single women who do not have a male partner as outlined above.

People wishing to have ART treatment in South Australia are required to sign a statutory declaration prior to commencing treatment to indicate that they do not have a criminal history that could impact on the health and welfare of a child to be born. Legislation introduced in Victoria in 2010 requires eligible women and their partners (if they have one) to undergo criminal record and child protection order checks prior to treatment and they are denied access to ART if they have convictions of violent or sexual offences or have had a child removed from their custody (16). Victoria is believed to be the first jurisdiction in the world to implement such rigorous checks as prerequisites for ART treatment.

### Surrogacy

#### Regulatory framework

For many years, surrogacy was legal only in the Australian Capital Territory and Victoria. In the last few years, other states and territories have changed their laws and regulations to allow altruistic surrogacy and this is now legal in most parts of Australia. Some states, such as Queensland and Tasmania, still prohibit surrogacy (42, 43). However, a surrogacy Bill is currently before the Queensland Parliament which, if passed, will allow surrogacy arrangements to occur in that state (44). Development of specific surrogacy legislation is also underway in New South Wales. Current provisions in the Assisted Reproductive Technology Act 2007 (NSW) only address the prohibition of commercial surrogacy and do not provide further guidance to clinics about the conduct of surrogacy arrangements (14). In addition, the Australian Government is currently considering harmonising surrogacy laws across Australia and conducted consultation on this matter in 2009 (45). The outcomes of this consultation are not yet known.

A number of restrictions apply to surrogacy in Australia. For example, surrogates are not able to use their own oocytes in a surrogacy arrangement. Instead, the commissioning woman’s oocyte or a donor oocyte may be used. In addition, commercial surrogacy, where a woman is paid to carry a pregnancy, is banned throughout Australia. However, as in other countries where only non-commercial surrogacy is legal (46), reimbursement of reasonable expenses is allowed and may include:

- Medical expenses associated with the pregnancy and birth that are not recoverable under Medicare or by private health insurance
- Cost of obtaining legal advice in relation to the surrogacy
- Cost of counselling associated with the surrogacy
- Travel costs associated with the pregnancy or birth
- Loss of earnings due to leave taken during the pregnancy or around the time of the birth
- Premiums payable for health, disability or life insurance that would not have been taken out if the surrogacy arrangement had not been entered into.

In Australia, the woman who gives birth to a child is presumed to be the child’s mother and her male partner, if she has one, is presumed to be the father. Provisions are in place in some Australian states to transfer parentage to the commissioning couple. This has been in place in ACT for some years, has recently been introduced in Victoria and WA, and will be introduced in South Australia by the end of 2010.

#### Informed decision making

In states with surrogacy legislation and under the NHMRC Ethical Guidelines, counselling is mandatory for all parties involved in a surrogacy arrangement. In some states, matters to be covered in counselling are prescribed and may include:

- Implications of surrogacy for the relationship between the commissioning parent/s, the surrogate and her partner (if she has one) and the donor, if donor gametes are used
- Implications of the surrogacy on existing children of the surrogate or commissioning parent/s
- Possibility of and attitudes of all parties toward prenatal screening and diagnosis, termination due to fetal genetic or chromosomal abnormalities or other pregnancy complications
- Possibility of any party deciding not to proceed
- Matters associated with the health of the fetus during the pregnancy
- Dispute resolution processes
- Commissioning parents’ intentions for care of the child should one of them die
- Possible grief reactions of the surrogate and her partner (if she has one)
- How to tell the child about the surrogacy
- Attitudes towards an ongoing relationship between the parties (17).

In all states with surrogacy legislation, participants are also required to obtain legal advice in relation to the surrogacy arrangement.

#### Surrogate and commissioning couple requirements

Eligibility requirements for surrogacy in the states where it can be undertaken vary. State laws and NHMRC Ethical Guidelines require that participants undergo counselling to consider the implications of the surrogacy arrangement for all parties and for the child to be born and that they understand the medical, legal, social and ethical implications of the surrogacy arrangement (13). Furthermore, surrogacy is only permitted when the commissioning female partner is unable to carry a pregnancy or give birth herself.

In addition, state legislation may specify a minimum age for surrogates, requirements for the surrogate to have previously carried a pregnancy and given birth, and requirements for obtaining legal advice in relation to the surrogacy arrangement and transfer of parentage to the commissioning parent/s. Both Victoria and Western Australia require the surrogate to be over 25 years of age. In some states, access to surrogacy is restricted to couples who are married or in a de facto relationship. In Western Australia, surrogacy is also available to single women who cannot conceive or give birth for medical or genetic reasons (47) and in Victoria, same-sex couples and single people (regardless of sex) are able to commission a surrogate (16).

## Conclusion

In Australia, the provision of gamete and embryo donation and surrogacy are regulated in state laws, the Fertility Society of Australia’s Reproductive Technology Accreditation Committee’s (RTAC) Code of Practice, and the National Health and Medical Research Council’s (NHMRC) Ethical Guidelines on the Use of Assisted Reproductive Technology in Clinical Practice and Research. The use of donor gametes and embryos and surrogacy to form a family is socially accepted and legal in most parts of Australia. The state of Victoria, as the first jurisdiction in the world, pioneered ART legislation and paved the way for other states that now have laws pertaining to the provision of ART services, including donor procedures and surrogacy. There are some minor differences between the state laws that govern third-party reproduction. However state laws, the RTAC Code of Practice and the NHMRC Ethical Guidelines are all built on the same three main principles:

- Commercial gamete and embryo donations and surrogacy arrangements are banned and hence third-party reproduction can only be undertaken for altruistic reasons.
- People considering donor procedures or surrogacy are required to undergo extensive counselling regarding the medical, social, psychological, ethical and legal complexities of third-party reproduction before proceeding.
- Donor-conceived children have a paramount right to be informed of their donor origins. Therefore, only donors who are willing to be identified when a donor-conceived child reaches the age of 18 can donate gametes and embryos and parents are strongly encouraged to disclose to their children that they were donor-conceived. To allow donors to be traced, ART service providers are obliged to maintain detailed records with identifying and non-identifying information about donors, recipients and children born as a result of donor procedures and, in some states, provide this information to state run donor registers.
